# Overview of Human Intervention Studies Evaluating the Impact of the Mediterranean Diet on Markers of DNA Damage

**DOI:** 10.3390/nu11020391

**Published:** 2019-02-13

**Authors:** Cristian Del Bo’, Mirko Marino, Daniela Martini, Massimiliano Tucci, Salvatore Ciappellano, Patrizia Riso, Marisa Porrini

**Affiliations:** 1 Department of Food, Environmental and Nutritional Sciences (DeFENS), Università degli Studi di Milano, 20122 Milan, Italy; mirko.marino@unimi.it (M.M.); massimiliano.tucci.mt@gmail.com (M.T.); salvatore.ciappellano@unimi.it (S.C.); patrizia.riso@unimi.it (P.R.); marisa.porrini@unimi.it (M.P.); 2 Human Nutrition Unit, Department of Veterinary Science, University of Parma, 43125 Parma, Italy; daniela.martini@unipr.it

**Keywords:** Mediterranean diet, DNA damage, DNA repair, telomere length, dietary intervention study

## Abstract

The Mediterranean diet (MD) is characterized by high consumption of fruits, vegetables, cereals, potatoes, poultry, beans, nuts, lean fish, dairy products, small quantities of red meat, moderate alcohol consumption, and olive oil. Most of these foods are rich sources of bioactive compounds which may play a role in the protection of oxidative stress including DNA damage. The present review provides a summary of the evidence deriving from human intervention studies aimed at evaluating the impact of Mediterranean diet on markers of DNA damage, DNA repair, and telomere length. The few results available show a general protective effect of MD alone, or in combination with bioactive-rich foods, on DNA damage. In particular, the studies reported a reduction in the levels of 8-hydroxy-2′–deoxyguanosine and a modulation of DNA repair gene expression and telomere length. In conclusion, despite the limited literature available, the results obtained seem to support the beneficial effects of MD dietary pattern in the protection against DNA damage susceptibility. However, further well-controlled interventions are desirable in order to confirm the results obtained and provide evidence-based conclusions.

## 1. Introduction

Oxidative stress is a condition characterized by an imbalance between formation of reactive oxygen species (ROS) and antioxidant defense mechanisms. Overproduction of ROS can cause oxidative damage to lipids, proteins, and DNA [1]. The integrity and stability of DNA is essential to life and for the maintenance of normal cell functions. The most common types of stressors, apart from oxidative species, include chemical agents, ultraviolet/ionizing radiation, and xenobiotics that can contribute to DNA damage and to formation of base deamination, base alkylation, base dimerization, base oxidation, and single/double strand breakage [1]. The resulting DNA damage, if not properly repaired, can increase risk of mutagenesis and bring to the onset or the development of numerous degenerative diseases including cardiovascular diseases (CVDs), diabetes mellitus, Alzheimer’s disease, and cancer [2,3,4]. Chronic oxidative stress has also been reported as a critical mechanism involved in telomere shortening [5]. Telomeres consist of long stretches of TTAGGG-DNA repeats associated with specific proteins and located at the end of chromosomes. They are involved in the protection of chromosomic stability and have been recognized as potential biomarkers of biological aging [6].

Increasing evidence suggests the crucial role of dietary and lifestyle habits as determinants of DNA oxidative damage [7,8], DNA repair [9], and telomere length [10]. Bioactives and bioactive-rich foods can exert a protective effect against oxidative stress resulting in lower DNA damage. For instance, DNA damage protection has been observed in several human intervention studies following the consumption of tomato [11,12], broccoli [13,14], spinach [15,16], blueberry [17,18], orange juice [19,20], nuts [21,22], green tea [23,24], and coffee [25,26]. Most of these foods are present in the Mediterranean diet (MD) and represent a rich sources of bioactive compounds such as vitamins, carotenoids, glucosinolates, and polyphenols acting as antioxidants or activators of endogenous multiple defense systems [27,28]. In addition, food bioactives have been demonstrated to induce DNA repair activity and to help in maintaining telomere length [10].

The MD has been identified as a sustainable and healthy dietary pattern characterized by a high intake of vegetables, legumes, fruits and nuts, cereals, olive oil, a moderately high intake of fish, a low-to-moderate intake of dairy products, a low intake of meat and poultry, and a regular but moderate intake of alcohol, primarily in the form of wine and generally within meals [29,30]. The adherence to the MD has been recognized to have a favorable effect on blood pressure, insulin sensitivity, lipid profile, inflammation, and oxidative stress, with a consequent decreased risk of numerous non-communicable diseases such as CVDs, cancer, and related deaths [31,32,33]. The results of PREDIMED (PREvención con DIeta MEDiterránea), a multicenter, randomized, nutritional intervention trial carried out in Spain from 2003 to 2011, demonstrated the protective effect of the traditional MD against CVD in individuals at high cardiovascular risk [34,35,36,37].

The exact mechanisms by which MD exerts its protective effects are not yet completely understood since numerous aspects such as lifestyle and environmental factors, characterizing Mediterranean style, can be involved and may interact contributing to the health benefits observed. However, the intake of bioactive compounds with a direct and/or indirect antioxidant action could represent a plausible, even if not the unique, explanation for the apparent benefits. A specific target of this protection could be represented by the defense of DNA from oxidative damage. The present review summarizes the main findings derived from human intervention studies addressing the impact of the MD on DNA protection and telomere length.

## 2. Materials and Methods

### 2.1. Search Strategy and Study Selection

**Figure 1:**
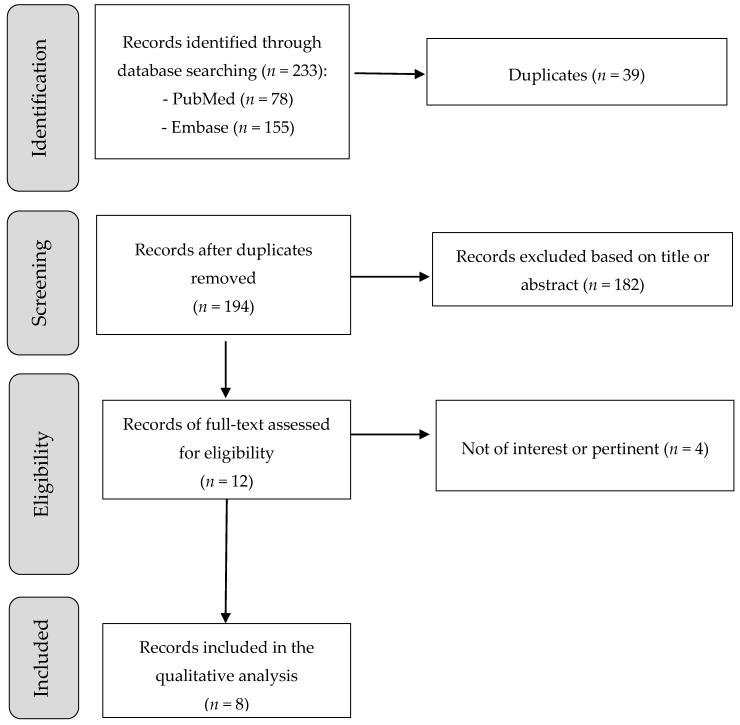
Flowchart of the study selection process.

PUBMED (http://www.ncbi.nlm.nih.gov/pubmed) and EMBASE (http://www.embase.com/) databases (updated December 2018) were searched to identify pertinent articles. The searches used the combination of the following terms: (Diet, Mediterranean OR Mediterranean diet OR diets, Mediterranean OR Mediterranean diets) AND (DNA damage OR DNA damages OR damage, DNA OR damages, DNA OR DNA injury OR DNA injuries OR injuries, DNA OR injury, DNA OR genotoxic stress OR genotoxic stresses OR stresses, genotoxic OR stress, genotoxic OR telomeric DNA damage OR telomere length). The retrieved papers were also screened for additional papers. The search strategy is summarized in Figure 1.

The search was limited to human intervention studies. No other specific restrictions for the selection of the studies have been used with the exception of the language. Only papers written in English have been considered. Two independent reviewers (MM and DM) conducted the literature search in the scientific databases and assessed and verified the eligibility of the studies based on the title and abstract. Disagreement between reviewers was resolved through consultation with a third independent reviewer (CDB) to reach a consensus.

Data extraction of the papers meeting the inclusion criteria was performed by two reviewers (MM and MT). The following information was reported: (1) first author name and year of publication; (2) the country where the study was performed; (3) study design; (4) subjects’ characteristics; (5) characteristics of the control group; (6) characteristics of the intervention group; (7) list of markers of DNA damage; and (8) main findings. 

### 2.2. Risk of Bias in Individual Studies

Risk of bias in individual studies was assessed independently by two review authors (MM and DM) by considering the following components to produce the resulting scores: (1) Selection Bias—Sequence generation and allocation concealment; (2) Performance Bias—Blinding of participants and personnel; (3) Detection Bias—Blinding of outcome assessment; (4) Attrition Bias—Incomplete outcome data; (5) Reporting Bias—Selective reporting; and (6) Other Bias. Scores were assessed considering three potential bias risks: “Low risk of bias”, when the study presented the considered characteristics; “High risk of bias”, when the study did not fully highlight the considered characteristics; and “Unclear risk of bias”, when it was not possible to attribute one of the two other scores (“Low risk” or “High risk”) due to missing information. All disagreements were resolved by consensus with a third reviewer (CDB).

## 3. Results and Discussion

### 3.1. Study Selection

A total of 233 records were identified from PubMed and EMBASE database search. After removing 39 duplicate articles, 194 studies were screened and 182 excluded based on title or abstract. The full text of eligible studies (*n* = 12) was read; a total of four records were excluded because not of interest or pertinent. At the end of the selection process a total of 8 human intervention studies were included in the review [38,39,40,41,42,43,44,45].

### 3.2. Study Characteristics

The main characteristics of the studies included in the review are reported in Table 1. Six out of eight studies were conducted in Spain [39,40,41,42,44,45], one in New Zealand [38], and one in Chile [43]. The study population included males and females of different age (young, adults and older subjects), and with different characteristics: healthy subjects [39,41], individuals with high cardiovascular risk [44,45], metabolic syndrome [40], and prostate cancer [38]. All the studies showed a randomized controlled design with the exception of one non-randomized and non-controlled study [38]. The duration of the intervention varied from a minimum of 4 weeks [39,41,43] up to 5 years [44,45]. Most of the investigations did not analyze the effects of MD alone but in combination with olive oil [40,42], nuts [40], wine [43], or MD supplemented with coenzyme Q10 [39,41]. Among the various markers of DNA oxidative damage, 8-hydroxy-2′-deoxyguanosine (8-oxo-dG) was the main evaluated, followed by the expression of DNA repair genes and telomere length. Only one study quantified the levels of strand breaks and oxidatively-induced DNA damage in peripheral blood mononuclear cells (PBMCs) [38].

### 3.3. Risk of Bias of the Studies

Risks of bias within individual studies are reported in Figures S1 and S2. On the whole, the results show an unclear risk of bias for most of the types of bias. The performance (blinding of participants and personnel) and the selection (i.e., random sequence generation) represent the highest risks of bias.

### 3.4. Main Findings

The characteristics of the intervention, the type of markers analyzed and the main findings of the studies are reported in Table 2. On the whole, the results obtained show that MD alone or in combination with specific bioactive-rich foods (i.e. olive oil, nuts, red wine) or antioxidant compounds (i.e., coenzyme Q10), may exert a protection against DNA damage and play an important role in the modulation of DNA repair genes (e.g. down-regulation of growth arrest and DNA-damage-inducible alpha, polymerase (DNA directed)). In particular, three studies reported a reduction of the levels of 8-oxo-dG in urine [40], plasma [41], or peripheral blood leukocytes [43] following the intervention with the MD alone or in combination with others foods/dietary components, while one study did not report a significant effect [42]. Conversely, one study showed a significant decrease in the levels of oxidatively-induced, but not endogenous, DNA damage [38]. Regarding DNA repair capacity, two studies showed a significant modulation of the genes involved in this repair pathway activity [39,41], while one study failed to report a significant effect [42]. The effect of MD on telomere length has been investigated within the PREDIMED-NAVARRA study. One paper did not show any effect on telomere length after MD intervention [44], while the other reported a significant increase in telomeres depending on different individual gene variants [45].

To the best of our knowledge, this is the first review aimed at providing evidence on the effects of MD in the modulation of DNA damage, DNA repair genes, and telomere length. The availability of data on protection from DNA damage is significant, since it has been reported to play a crucial role in the development of degenerative diseases, while the adherence to MD can represent a protective dietary pattern.

It is noteworthy that most of the studies focused their attention on the dietary fat quality as possible determinant of DNA damage and numerous diseases. In particular, it has been suggested that the amount and quality of dietary fats could be important for the maintenance of DNA stability. Some researchers found that a polyunsaturated fatty acid (PUFA)-rich diet was associated with reduced DNA damage [46], while saturated fatty acid (SFA) intake was demonstrated to increase DNA damage [47]. In this regard, a recent study evaluated the association between fat intake, as part of a modified Mediterranean style dietary intervention study, and several markers of inflammation and oxidative stress including DNA damage [48]. The authors found an inverse correlation between DNA damage and monounsaturated fatty acids (MUFAs), particularly oleic acid, while a positive correlation was observed between DNA damage and the intake of dairy products and red meat (possibly due to SFAs) [48].

The role of MUFAs and PUFAs in the modulation of the levels of DNA damage and DNA repair has been poorly investigated in vivo and results, in particular for PUFA, are quite controversial. For example, Bishop et al. [48] found a positive correlation between DNA damage and circulating levels of omega-6 PUFA. Similar findings were found in other in vivo studies hypothesizing a process of lipid peroxidation as determinant of DNA damage [49,50,51]. Conversely, numerous other studies have reported a protection of MUFAs and PUFAs against oxidative stress and DNA damage [46,52,53]. In the present review, the role of olive oil and nuts as food sources of MUFA and PUFAs respectively, but not limited to them, has been evaluated within a context of Mediterranean diet. Mitjavila and colleagues [40] performed a randomized, controlled, parallel clinical trial in which 110 female subjects with metabolic syndrome, recruited within the PREDIMED study, were randomly assigned to three intervention groups: (1) MD *plus* nuts; (2) MD *plus* extra virgin olive oil; and (3) control diet (advice on low-fat diet). The effects of these three interventions on the levels of DNA damage and other parameters related to cardiovascular health were evaluated after 1-year follow-up. The results obtained showed an overall significant improvement of cardiovascular health outcomes and a reduction in the urinary levels of 8-OH-dG in the MD group as compared to the control group. In another study, Konstantinidou and coworkers [42] investigated the effects of the traditional MD, associated with the consumption of two different virgin olive oils, on the expression of atherosclerosis markers and related genes including DNA damage and DNA repair genes. To this aim, the authors performed a randomized, parallel, controlled clinical trial with three different interventions: (1) MD *plus* virgin olive oil; (2) MD *plus* washed virgin olive oil (low content in polyphenols compared to normal virgin olive oil); (3) control diet consisting in the habitual diet of the participants. The impact of the dietary interventions was evaluated in 90 subjects (30 subjects for each arm of intervention) after 3 months. The results have shown an overall general improvement of the MD intervention on the genes and markers related to atherosclerosis, while the effect on the urinary levels of 8-OH-dG was significant only after the intervention with virgin olive oil but not washed virgin olive oil. Conversely, the effect on the modulation of DNA repair genes was significant only when considering the global effect of both the interventions with the MD and olive oil. The results showed a down-regulation of polymerase (DNA directed) k (POLK) gene expression, suggesting a protective role of the MD on DNA oxidation and damage. In fact, POLK is a specialized DNA polymerase that catalyzes the translesion DNA synthesis, which allows DNA replication in the presence of lesions. The authors attributed the beneficial effects of the intervention to presence of MUFAs and other components (i.e. polyphenols) provided by olive oil and the MD.

SFAs may promote cell transformation by negatively regulating the DNA damage response pathway [49]. Gutierrez-Mariscal et al. [41] evaluated the impact of a 4-week intervention with MD and MD *plus* coenzyme Q10 (a fat-soluble bioactive with antioxidant activity), compared to a SFA-rich diet, on different markers of DNA damage and DNA repair in a group of elderly subjects. The authors found a significant decrease in p53 protein levels (a transcription factor which mediates the cellular response to DNA damage), as well as plasma circulating 8-OH-dG levels (as marker of DNA damage), following both the Mediterranean dietary approaches but not with the diet rich in SFAs. The authors also demonstrated, in the same population, that the adherence to MD was able to control the expression of DNA repair genes, while the intervention with the SFA diet triggered the p53-dependent DNA repair machinery, as defense mechanism in response to a stress condition [26]. Similarly, Urquiaga et al. [43] investigated the effect of a 3-month MD intervention versus an Occidental diet (OD, resembling a Western or U.S. diet) richer in fat, in particular SFAs, in a group of young healthy subjects. During the first and the third month, subjects received the prepared diets alone, while during the second month they were asked also to add 240 mL of red wine (corresponding to 2 glasses per day). Subjects on MD, compared to those on OD (higher in SFAs), had reduced levels of 8-OH-dG in peripheral blood leucocytes. However, since both the diets included a moderate consumption of red wine for a period of 30 days, a positive or negative contribution of this product in the results obtained cannot be excluded.

DNA damage has been positively associated with cancer risk. In fact, it has been clearly documented that DNA lesions can alter the primary structure of the double helix thereby affecting transcription and replication [53]. Moreover, erroneous DNA repair of lesions can lead to mutations or chromosomal aberrations affecting oncogenes and tumor suppressor genes; the effect is that cells undergo malignant transformation resulting in cancerous growth with deleterious consequences for individual’s health [53]. Several epidemiological studies reported an inverse association between MD adherence and neoplastic diseases [54,55,56] at the level of different sites such as breast [57], colorectal [58], bladder [59], and prostate cancer [60]. In the present review, we included a dietary intervention study performed on 20 subjects with prostate cancer in which the effect of a modified Mediterranean-style diet, consisting in extra-virgin olive oil, fresh frozen salmon, unsweetened pure pomegranate juice and canned legumes, was evaluated [38]. The intervention was a 3-month non-randomized controlled trial in which the levels of DNA damage in individual cells were evaluated by the comet assay [61,62]. The authors documented that dietary changes towards a modified Mediterranean-style pattern could have beneficial effects in terms of reduction of oxidatively-induced DNA damage in this specific target group [38]. This effect was attributed to an undeterminable synergistic effect of dietary components provided through the MD intervention.

Some observational and ex-vivo studies have shown that a greater adherence to MD was associated not only with an improvement of health status, but also with longer telomeres and higher telomerase activity in the elderly [63,64,65,66]. Garcia-Calzόn and colleagues [44] found that a higher baseline adherence to the MD was associated with longer telomerases in women, but not in men, from the PREDIMED-NAVARRA study. After the 5-year intervention, the results obtained showed that the MD failed to prevent telomere shortening in this target group of older subjects. However, further data elaboration, by considering some gene variants (i.e., Pro/Ala polymorphisms of the peroxisome proliferator-activated receptor γ2), showed that subjects with Ala carrier variant had increased telomeres length following MD intervention [45]. Moreover, the research team documented that the adherence to MD intervention improved obesity parameters [67] and dietary inflammatory index [68], likely slowing down telomere shortening.

## 4. Conclusions

Undoubtedly, the MD represents a balanced and healthy dietary pattern associated with a reduced risk of major chronic diseases. However, the role of the MD in the modulation of DNA damage, as potential contributor in disease onset, is poorly investigated. Apart from PREDIMED, most of the studies did not focus on the role of the MD as such, but in combination with specific bioactives and/or bioactive-rich foods; thus, specific contributions are difficult to discern. Although preliminary, the papers reviewed seem to strengthen the hypothesis of a potential role of MD in the protection of DNA damage and in the modulation of DNA repair genes and telomere length. However, the studies show several limitations due to the numerous bias and confounding factors apparently present in the experimental designs. For these reasons, the results in the present review can represent a starting point for further well-controlled intervention studies targeted on the specific effects of traditional and/or revised MD pattern in the modulation of DNA damage. These studies will be useful to provide more evidence-based proof of MD protective activity and to reveal the molecular mechanisms at the base of the protection observed.

## Figures and Tables

**Table 1:** Characteristics of the studies

Reference	Country	Subject Characteristics	Study Design	Control
Erdrich et al. [38]	New Zealand	Subjects = 20 (F = 0; M = 20) with prostate cancer, Age = 52–74 years; BMI = 23–33 kg/m^2^ Non-smokers = 7; former-smokers = 13	One arm intervention	N.A.
Gutierrez-Mariscal et al. [39]	Spain	Subjects = 20 (F = 10; M = 10); Age = > 65 years; BMI = 20–40 kg/m^2^ Non-smokers = 20	Randomized, controlled crossover trial	Western diet rich in SFA (6 subjects)
Mitjavila et al. [40]	Spain	Subjects = 110 (F = 110; M = 0) with MetS; Age = 55–80 years; BMI = < 35 kg/m^2^ Non-smokers	Multicentric, randomized, controlled, parallel clinical trial PREDIMED	Low-fat diet (37 subjects)
Gutierrez-Mariscal et al. [41]	Spain	Subjects = 20 (F = 10; M = 10); Age = > 65 years; BMI = 20–40 kg/m^2^ Non-smokers=20	Randomized, controlled crossover trial	Western diet rich in SFA (6 subjects)
Konstantinidou et al. [42]	Spain	Subjects = 90 (F = 64; M = 26); Age = 20–50 years; BMI = < 30 kg/m^2^ Smokers = N.A.	Randomized, parallel, controlled clinical trial	Habitual diet (30 subjects)
Urquiaga et al. [43]	Chile	Subjects = 42 (F = 0; M = 42); Age = 20–27 years; BMI = N.A. Smokers = N.A.	Partially randomized, controlled, trial	Occidental diet (21 subjects)
García-Calzon et al. [44]	Spain	Subjects = 520 (F = 286; M = 234) at high CV risk; Age = 55–80 years; BMI = 25–35 kg/m^2^ Non-smokers = 326; former-smokers = 117; smokers = 77	Multicentric, randomized, controlled, parallel clinical trial PREDIMED-NAVARRA	Low-fat diet (140 subjects)
García-Calzon et al. [45]	Spain	Subjects = 521 at high CV risk Pro/pro group = 451 (F = 244; M = 207); Ala carrier group = 70 (F = 64; M = 25) Age = 60–80 years F, 55–80 years M; BMI = 25–35 kg/m^2^	Multicentric, randomized, controlled, parallel clinical trial PREDIMED-NAVARRA	Low-fat diet (140 subjects)

Legend: BMI: body mass index; CV: cardiovascular; EVOO: extra virgin olive oil; F: female; M: male; MD: Mediterranean diet; MetS: metabolic syndrome; N.A: not available; PREDIMED: PREvención con DIeta MEDiterránea project; SFA: saturated fatty acid.

**Table 2:** Main findings about the role of Mediterranean diet in the modulation of markers of DNA damage, DNA repair, and telomere length.

Reference	Intervention	Markers of DNA Damage	Main Findings
Erdrich et al. [38]	Adherence to Mediterranean-style diet consisting of: extra virgin olive oil, fresh frozen salmon (200 g/week), unsweetened pure pomegranate juice (1 L/week) and samples of a variety of canned legumes 3 months	Percentage DNA in the tail	↓cell DNA damage (*p* = 0.013)
Gutierrez-Mariscal et al. [39]	MD supplemented with Q10 (7 subjects) Only MD (7 subjects) 4 weeks Each diet	Gadd45a Ogg1 APE-1/Ref-1 DNA pol β XPC	↓ Gadd45a mRNA levels MD *plus* Q10 *vs.* SFA (*p* = 0.044) ↓ nuclear Gadd45a in fasting and at 4 h MD vs. SFA (*p* = 0.023 and *p* = 0.038, respectively) ↑ Ogg1 mRNA levels during postprandial period SFA *vs.* MD *plus* Q10 (*p* = 0.048) ↑ nuclear APE-1/ Ref-1 protein level during the postprandial period and long-term consumption SFA *vs*. MD *plus* Q10 (*p* = 0.038 and *p* = 0.028, respectively) ↓ DNApolβmRNA levels MD *plus* Q10 *vs.* SFA (*p* = 0.041) ↑ nuclear DNApolβ protein levels SFA vs. MD *plus* Q10 (*p* = 0.044) ↑ XPC mRNA levels during postprandial period SFA vs. MD *plus* Q10 (*p* = 0.019)
Mitjavila et al. [40]	MD *plus* EVOO (38 subjects) MD *plus* nuts (35 subjects) 1 year	8-OH-dG	↓ Urinary 8-OH-dG concentrations MD groups vs. Control (*p* < 0.001)
Gutierrez-Mariscal et al. [41]	MD supplemented Q10 (7 subjects) Only MD (7 subjects) 4 weeks	8-OH-dG P53 p-p53 (Ser20) p53R2	↓ 8-OH-dG plasma concentrations MD and MD *plus* Q10 vs. SFA (*p* < 0.0001) ↓ 8-OH-dG plasma concentrations MD *vs.* MD *plus* Q10 (*p* < 0.001) ↓ postprandial levels of cytoplasmic p53 MD *plus* Q10 *vs.* SFA and MD (*p* < 0.05) ↓ nuclear p-p53 (Ser20) postprandial levels MD *plus* Q10 *vs.* SFA and MD (*p* = 0.0013). ↑ p53 mRNA levels postprandial and after 2 h SFA vs. MD (*p* = 0.047). ↔ mRNA p53R2 MD *plus* Q10 vs. SFA vs. MD (*p* > 0.05)
Konstantinidou et al. [42]	MD *plus* VOO (30 subjects) MD *plus* WOO (30 subjects) 3 months	8-OH-dG CCNG1 POLK TP53 DCLRE1C DNA ERCC5 XRCC5	↓ polymerase (DNA directed)- (POLK) MD vs. control group (p <0.05) ↔ 8-OH-dG, CCNG1, TP53, DCLREIC, ERCC5, XRCC5 MD vs. control group (p >0.05)
Urquiaga et al. [43]	MD (21 subjects) 3 months	8-OH-dG	↓ 8-OH-dG in DNA from peripheral blood leukocytes MD group vs. OD group (p <0.008)
García-Calzòn et al. [44]	MD *plus* EVOO (210 subjects) MD *plus* nuts (170 subjects) 5 years	Telomere length	↔ telomere length MD *plus* EVOO vs. Control ↓ telomere length MD *plus* nuts vs. Control (*p* < 0.001)
García-Calzòn et al. [45]	MD *plus* EVOO (212 subjects) MD *plus* nuts (169 subjects) 5 years	Telomere length	↑ telomere length Ala carriers group *plus* MD vs. Pro/pro group *plus* MD (*p* < 0.01)

Legend: APE-1/Ref-1: Reduction-oxidation factor 1-apurinic/apyrimidinic endonuclease; CCNG1: Cyclin G1; DCLRE1C: DNA cross-link repair 1C; DNA pol β: DNA polymerase beta; EVOO: extra virgin olive oil; ERCC5: excision repair cross-complementing rodent repair deficiency, complementation group 5; Gadd45a: growth arrest and DNA-damage-inducible alpha; MD: Mediterranean diet; OD: Occidental diet; Ogg1: 8-oxoguanine DNA glycosylase; P53: protein 53; p-p53 (Ser20): pospho-p53 (serine20); p53R2: p53 inducible ribonucleotide reductase gene; POLK: polymerase (DNA directed); Q10: Coenzyme Q10; SFA: saturated fatty acids; TP53: tumor protein p53; VOO: virgin olive oil; WOO: washed virgin olive oil; XRCC5: X-ray repair complementing defective repair in Chinese hamster cells 5 (double-strand-break rejoining; Ku autoantigen, 80 kDa); XPC: xeroderma pigmentosum, complementation group C; 8-OH-dG: 8-hydroxy-2′–deoxyguanosine.

## Supplementary Materials

The following are available online at http://www.mdpi.com/2072-6643/11/2/391/s1, Figure S1: Risk of bias graph: review authors’ judgements about each risk of bias item presented as percentages across all included studies, Figure S2: Risk of bias summary: review authors’ judgements about each risk of bias item for each included study.
