# Supplementary figures and images for: Overview of Human Intervention Studies Evaluating the Impact of the Mediterranean Diet on Markers of DNA Damage
Source: Nutrients. 2019 Feb 13;11(2):391. doi: 10.3390/nu11020391 (PMC6412605; doi:10.3390/nu11020391)

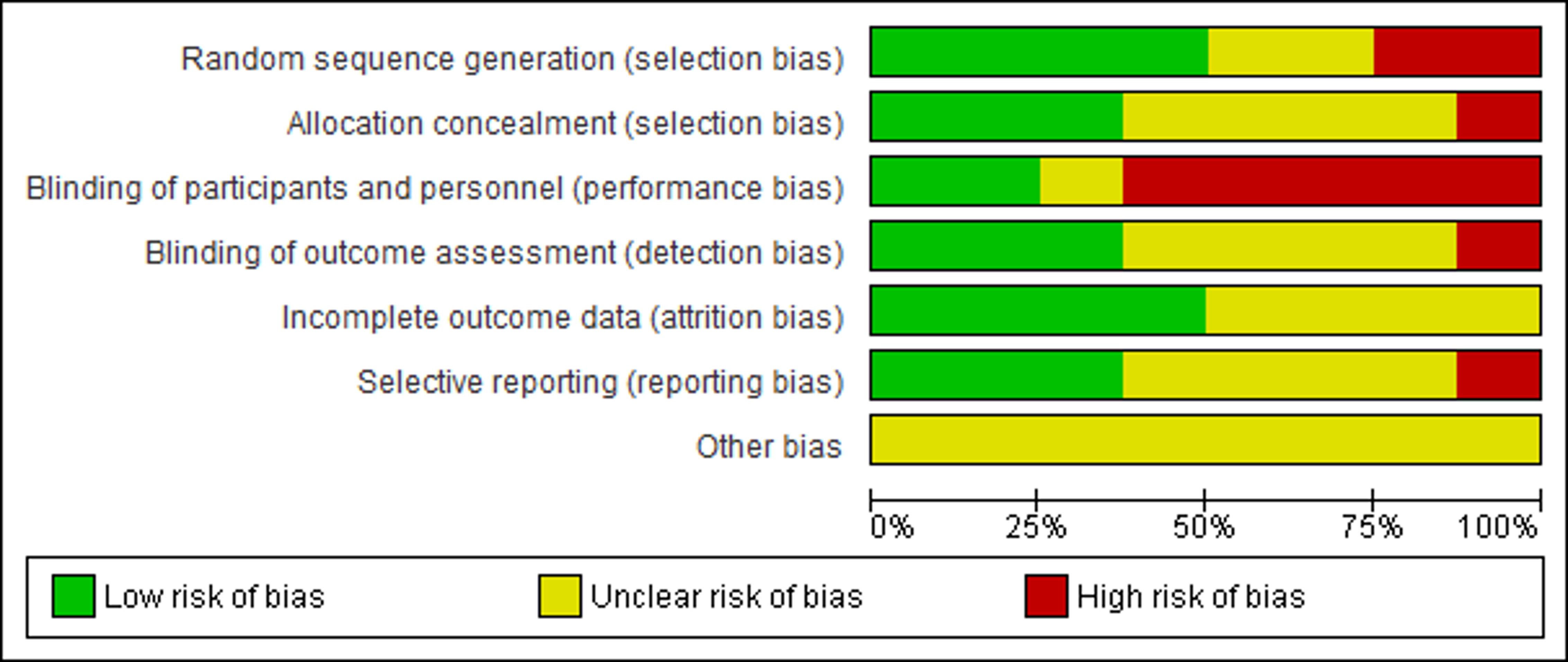

Supplement: Supplementary file 1 [file nutrients-11-00391-s001.zip › Revised Supplementary materials/Suppl1.tif]

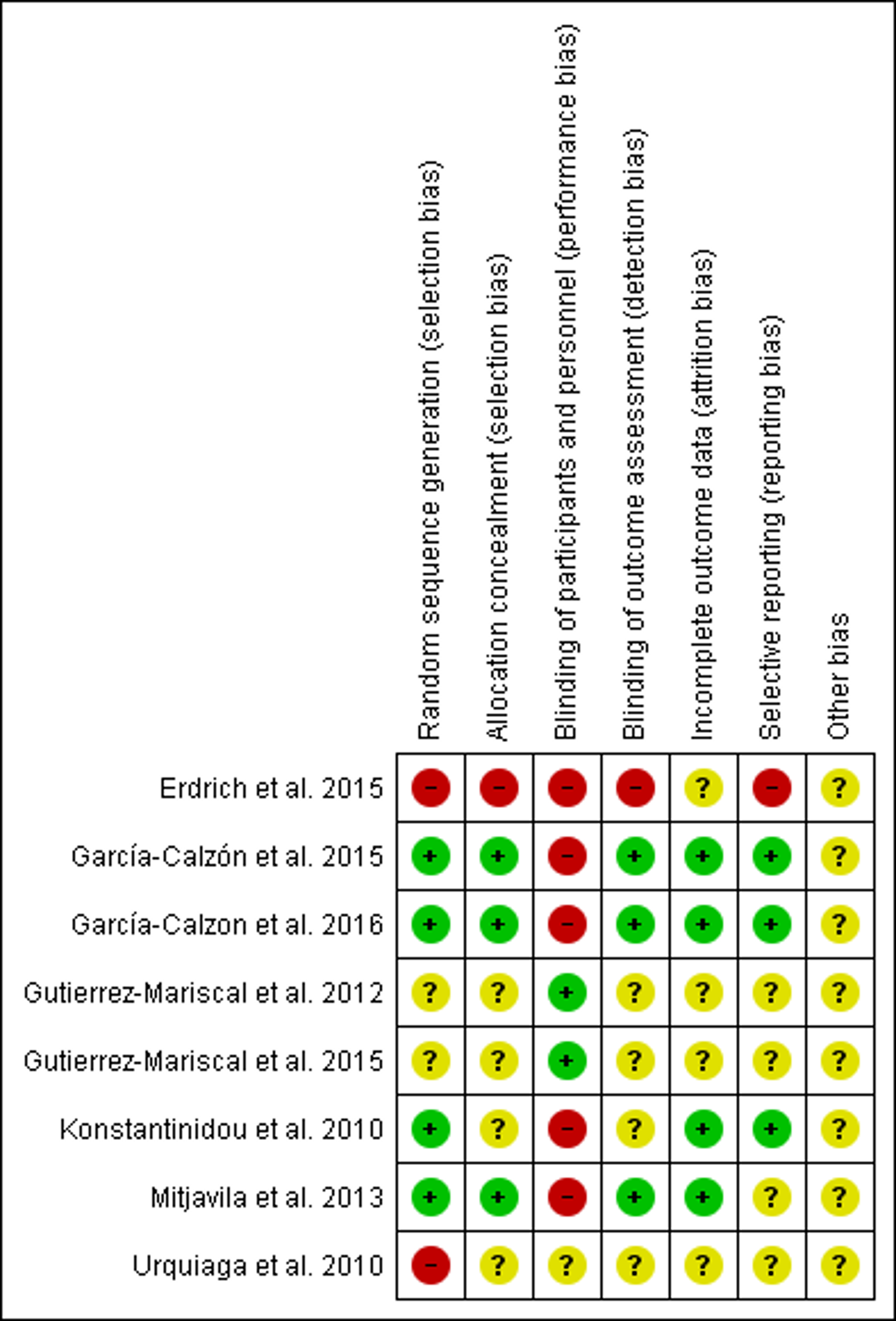

Supplement: Supplementary file 1 [file nutrients-11-00391-s001.zip › Revised Supplementary materials/Suppl2.tif]
